# The outer membrane protease PgtE of *Salmonella enterica* interferes with the alternative complement pathway by cleaving factors B and H

**DOI:** 10.3389/fmicb.2015.00063

**Published:** 2015-02-06

**Authors:** Rauna Riva, Timo K. Korhonen, Seppo Meri

**Affiliations:** ^1^Immunobiology Research Program, Research Program Unit, University of HelsinkiHelsinki, Finland; ^2^Department of Bacteriology and Immunology, Haartman Institute, University of HelsinkiHelsinki, Finland; ^3^General Microbiology, Department of Biosciences, University of HelsinkiHelsinki, Finland; ^4^HUSLAB, Hospital District of Helsinki and UusimaaHelsinki, Finland

**Keywords:** *Salmonella*, protease, phagocytosis, immune evasion, PgtE, complement factor B, complement factor H, omptin

## Abstract

The virulence factor PgtE is an outer membrane protease (omptin) of the zoonotic pathogen *Salmonella enterica* that causes diseases ranging from gastroenteritis to severe enteric fever. It is surface exposed in bacteria that have a short-chain, i.e., rough LPS, as observed e.g., in bacteria residing inside macrophages or just emerging from them. We investigated whether PgtE cleaves the complement factors B (B) and H (H), key proteins controlling formation and inactivation of the complement protein C3b and thereby the activity of the complement system. *S. enterica* serovar Typhimurium or omptin-expressing recombinant *E. coli* bacteria were incubated with purified human complement proteins or recombinant H fragments. PgtE cleaved both B and H, whereas its close homolog Pla of *Yersinia pestis* cleaved only H. H was cleaved at both N- and C-termini, while the central region resisted proteolysis. Because of multiple effects of PgtE on complement components (cleavage of C3, C3b, B, and H) we assessed its effect on the opsonophagocytosis of *Salmonella*. In human serum, C3 cleavage was dependent on proteolytically active PgtE. Human neutrophils interacted less with serum-opsonized FITC-stained *S. enterica* 14028R than with the isogenic Δ*pgtE* strain, as analyzed by flow cytometry. In conclusion, cleavage of B and H by PgtE, together with C3 cleavage, affects the C3-mediated recognition of *S. enterica* by human neutrophils, thus thwarting the immune protection against *Salmonella*.

## Introduction

*Salmonella enterica* serovar Typhi causes typhoid fever, a systemic disease with a global annual burden of 27 million cases and a case-fatality rate of 1%. In contrast to the human-restricted Typhi, most *S. enterica* serovars are zoonotic pathogens. Indeed, salmonellosis is the second most common zoonosis in Europe, after *Campylobacter* infections. While in humans the serovar Typhimurium causes mainly a self-limited gastroenteritis, in susceptible mouse strains it causes a disease similar to human typhoid fever. It can cause a systemic disease also in humans. Notably, it is the leading serotype causing invasive non-typhoidal salmonellosis in Africa (Crump et al., 2004; Buckle et al., 2010; Graham, 2010; Wick, 2011; Eurosurveillance Editorial Team, 2012).

The complement system is in the front line of immune defense against invading microbes. It comprises ~50 proteins present in plasma or on cell surfaces. A contact with bacteria activates a cascade of serine protease reactions initiated by the alternative (AP), classical or lectin pathway. These pathways converge when C3 is activated by cleavage into C3a and C3b. C3b can bind covalently onto the bacterial surface, where it promotes phagocytosis as a key opsonin. Importantly, it nucleates the exponential amplification of complement cascade via AP. C3b can associate with the pro-enzyme factor B (B) that gets proteolytically activated by factor D into active Bb. The C3bBb complex, i.e., AP C3-convertase, generates more C3b molecules by liberating the anaphylatoxic peptide C3a from the C3α chain of C3. Ultimately, complement activation leads to the formation of membrane attack complex that disrupts the outer membranes of Gram-negative bacteria to induce osmotic lysis (Torreira et al., 2009; Ricklin et al., 2010). The self-amplifying property of complement warrants tight regulation to avoid excessive inflammation and damage to bystander host cells. Factor H (H) is a single-chain ~155 kDa glycoprotein that consists of 20 homologs short consensus repeat (SCR) domains, aka complement control protein (CCP) domains. Factor H regulates complement activity by three means. It is the main soluble co-factor for factor I-mediated inactivation of C3b into iC3b that is the preferred ligand for the phagocytic CR3 (CD11b/CD18) receptor. Factor H also inhibits the assembly and accelerates the decay of the AP C3-convertase C3bBb (Bajic et al., 2013; Makou et al., 2013). Many pathogens recruit H onto their surfaces to inhibit complement attack (Blom et al., 2009; Meri et al., 2013).

PgtE of *S. enterica* and Pla of *Yersinia pestis* that causes plague, belong to the family of enterobacterial outer membrane proteases called omptins. Omptins are structurally homologs ~70 Å β-barrel-folded transmembrane proteins exposing five loops on the bacterial surface. The omptins have a highly conserved catalytic groove and they cleave substrates after basic amino acids. However, the amino acid sequences as well as the lengths of the surface loops in omptins from different bacterial species are variable and dictate the polypeptide substrate specificity of individual omptins (Kukkonen et al., 2001; Ramu et al., 2008; Haiko et al., 2010; Korhonen et al., 2013). The omptins are unique surface proteases as they require short-chain, i.e., rough, LPS for their enzymatic activity toward polypeptide substrates (Kukkonen et al., 2004; Lahteenmaki et al., 2005; Eren et al., 2010; Eren and van den Berg, 2012). *Salmonella* is a facultatively intracellular bacterium residing mainly in permissive macrophages, where it expresses a shortened O-antigen and active PgtE (Lahteenmaki et al., 2005). Pla is active on the surface of *Y. pestis*, which inherently has a rough LPS type due to frameshift mutations within the O-antigen biosynthesis gene cluster (Skurnik et al., 2000). Also, *Y. pestis* modifies its LPS structure upon transfer from the flea temperature to the human temperature to favor enzymatic activity of Pla (Suomalainen et al., 2010) and to suppress innate immune responses to LPS (Montminy et al., 2006). In contrast to *Y. pestis*, *S. enterica* mainly spreads within the host as an intracellular pathogen that expresses rough LPS during its growth within or immediately after release from phagocytes (Eriksson et al., 2003; Lahteenmaki et al., 2005). *In vivo* studies in mouse models have established that both PgtE and Pla contribute to pathogenesis. Deletion of *pgtE* reduces the growth in macrophages and the systemic dissemination of *Salmonella* Typhimurium into liver and spleen by tenfold after intraperitoneal inoculation (Lahteenmaki et al., 2005; Ramu et al., 2008). Pla is central for the establishment of pneumonic and bubonic plague, and deletion of the *pla* gene attenuates *Y. pestis* in bubonic plague by millionfold (Sodeinde et al., 1992; Sebbane et al., 2006; Lathem et al., 2007).

Several complement proteins are omptin substrates. We previously reported that PgtE cleaves C3, C3b, C4, C4b, and C5 (Ramu et al., 2007). Pla cleaves C3 but is inactive against factor B (Sodeinde et al., 1992). Cpa, the omptin of the invasive pathogen *Cronobacter sakazakii*, cleaves C3, C3a, and C4b (Franco et al., 2011). Haiko and co-workers reported that PgtE and Pla cleave vitronectin that inhibits the membrane attack complex (Podack and Muller-Eberhard, 1979; Haiko et al., 2010). Here we investigated, whether the central complement factors B and H could be cleaved by PgtE and selected omptins of *Yersinia* and *E. coli*. Furthermore, since human serum contains several PgtE substrates and since the aforementioned studies have been conducted with purified complement proteins, we deemed it crucial to confirm the PgtE-mediated degradation of C3 in human serum. Finally, since factors B and H have opposing effects on the formation of C3b, the key opsonin, it was important to test how PgtE affects the opsonophagocytic uptake of *Salmonella* into human neutrophils.

## Results

### Cleavage of factors B and H by *Salmonella* is PgtE-dependent

**Figure 1:**
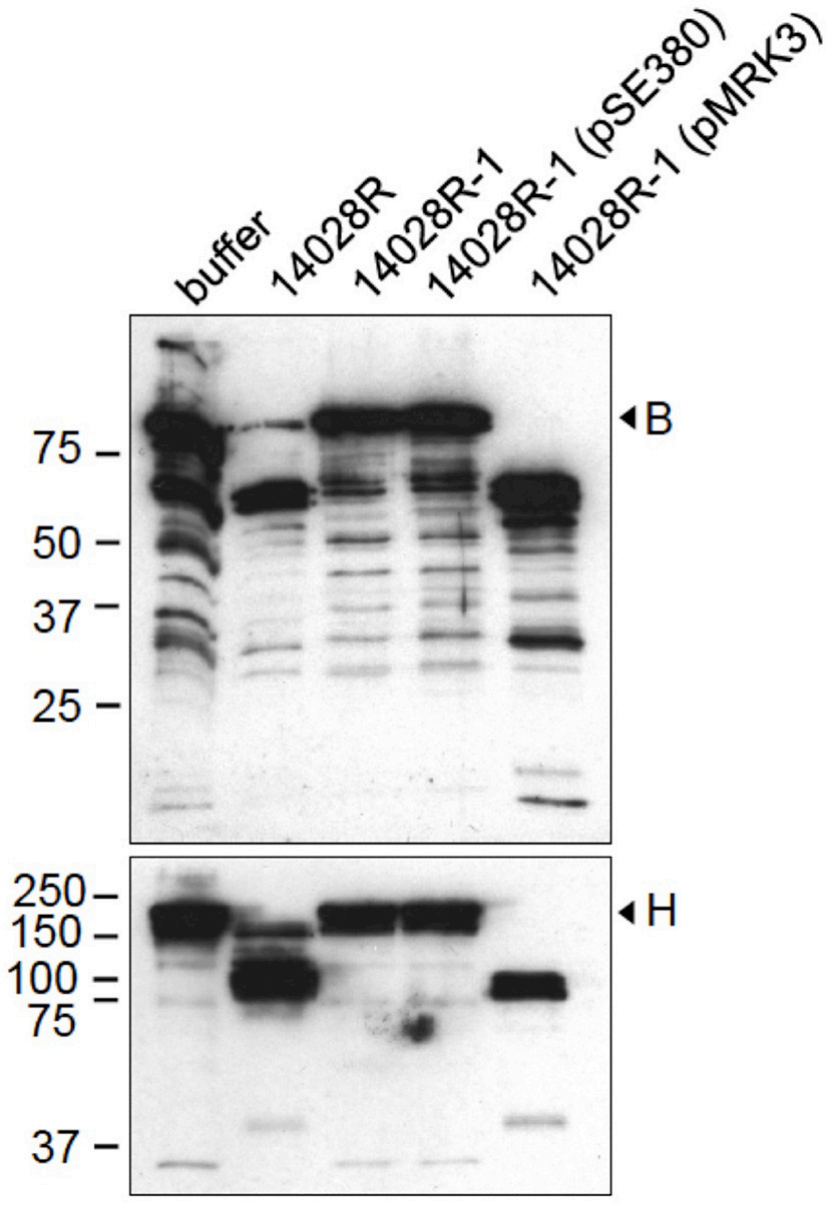
**Cleavage of factors B and H by *S. enterica* is PgtE-dependent**. *S. enterica* 14028R, its Δ*pgtE*-derivative 14028R-1 and PgtE (pMRK3)- or empty vector (pSE380) -complemented 14028R-1 strains were incubated with factors B or H (33 μg/ml) for 4 h. The reduced supernatants were immunoblotted with polyclonal antibodies against factors B or H. The assays were conducted at least twice with consistent results.

We first analyzed whether the virulent strain 14028 of *S. enterica* has the capacity to cleave the complement proteins B and H. Factor B is central to complement activity as factor D-activated Bb is the protease part of the C3-convertases C3(H_2_O)Bb and C3bBb (Torreira et al., 2009). B contains SCR-modules homologs to those in the regulatory factor H, and it has an opposite, complement activation promoting effect (Milder et al., 2007). For two reasons, the degradation of factors B and H was analyzed using the rough variant 14028R that is resistant to a smooth LPS-specific bacteriophage P22c2 (Wick et al., 1994). Enterobacterial omptins require rough LPS for enzymatic activity, and O-chains, which are typically long in *in vitro*-grown Typhimurium, sterically inhibit proteolysis (Kukkonen et al., 2004). *S*. Typhimurium 14028 modifies its LPS into a rough form during infection. Both the activity and expression levels of the PgtE protease of 14028 are high in cells isolated from macrophages (Eriksson et al., 2003; Lahteenmaki et al., 2005). Thus, the rough strain 14028R grown in *pgtE*-inducing medium mimics phagocyte-released *S. enterica*. The strain 14028R cleaved both B and H, whereas the Δ*pgtE* derivative 14028R-1 did not exhibit these activities (Figure 1). Complementation of 14028R-1 with *pgtE*-encoding pMRK3 recovered the activity toward B and H, whereas the introduction of the empty vector plasmid had no effect. Thus, PgtE was required for the cleavage of B and H by *Salmonella*.

**Figure 2:**
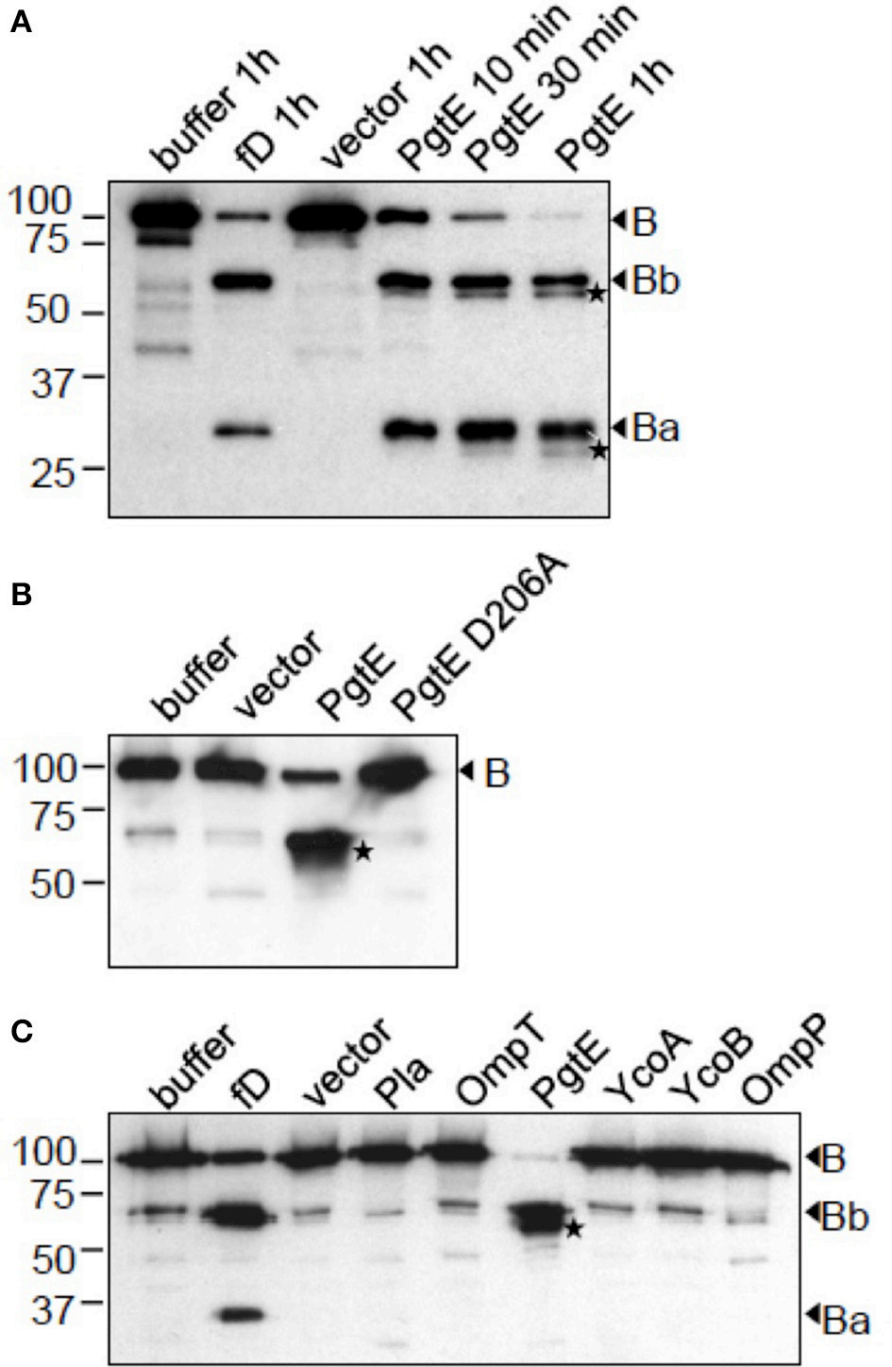
**Cleavage of factor B by recombinant *E. coli* expressing different omptin genes**. The omptin proteins are indicated above the lanes. Control reactions of B incubated in buffer, with the empty vector plasmid-carrying strain or with factor D in the presence of C3 and Mg^2+^, are shown for comparison. **(A)** *E. coli* 83972 Δ*ompT* bacteria expressing PgtE were incubated for 10 min, 30 min or 1 h with B (16.7 μg/ml). **(B)** *E. coli* XL1 bacteria expressing PgtE, D206A-mutant or the empty vector were incubated for 1 h with B (33 μg/ml). **(C)** *E. coli* 83972 Δ*ompT* bacteria expressing omptin proteins or the empty vector were incubated for 1 h with B (33 μg/ml). Non-reduced **(A)** or reduced **(B,C)** supernatants were immunoblotted with a polyclonal anti-B antibody. Factor D-generated cleavage products Bb and Ba are indicated. PgtE-specific cleavage products are marked with asterices (^*^). The assays were conducted at least twice with consistent results.

To confirm the role of PgtE in the degradations, we expressed *pgtE* in *E. coli* 83972Δ*ompT*, which is deleted for the endogenous omptin gene *ompT* and lacks *ompP* (Järvinen et al., 2013). *E. coli* 83972Δ*ompT* expressing PgtE cleaved B in a time-dependent fashion generating four immunoreactive fragments (Figure 2A). In non-reducing conditions the apparent sizes of the major fragments corresponded to those of the fragments Bb and Ba generated by factor D. PgtE generated also B fragments of approximately 57 and 28 kDa. However, when the samples were run under reducing conditions (Figures 2B,C) the PgtE-generated cleavage pattern of factor B was different. The Ba-sized or the 28 kDa-sized fragment could not be detected indicating cleavages at multiple sites. The different cleavage patterns are apparently due to intramolecular disulfide bridges that keep the proteolytically cleaved fragments together under non-reducing conditions. No other omptins, examined as controls, were able to cleave factor B (Figure 2C).

**Figure 3:**
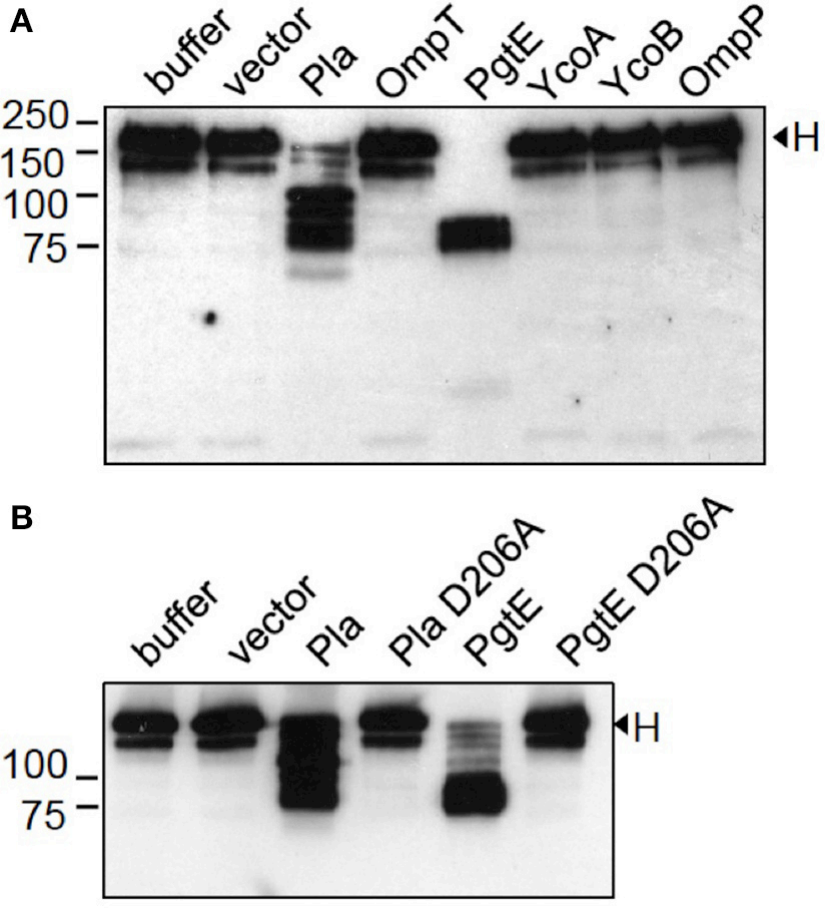
**Cleavage of factor H by *E. coli* expressing omptin proteins**. **(A)** *E. coli* 83972 Δ*ompT* bacteria expressing omptin proteins or the empty vector were incubated with purified H (33 μg/ml) for 4 h. **(B)** *E. coli* XL1 bacteria expressing Pla, PgtE, their D206A-mutants or the empty vector were incubated with H for 1 h. The supernatants were run in SDS-PAGE under reducing conditions and immunoblotted with a polyclonal anti-H antibody. Samples of H incubated in buffer are shown for comparison. The assays were conducted at least twice with consistent results.

*E. coli* 83972Δ*ompT* (Figure 3A) and *E. coli* XL1 (Figure 3B) expressing PgtE cleaved factor H at multiple sites resulting in ladder-like cleavage patterns. In comparison, *Y. pestis* Pla cleaved factor H less efficiently compared to PgtE, since more intact protein was left after incubation with the Pla-expressing strains. Previous reports have identified Asp206 as a catalytic amino acid in omptins (Kukkonen et al., 2001; Lahteenmaki et al., 2005; Eren et al., 2010; Haiko et al., 2010; Järvinen et al., 2013). *E. coli* XL1 bacteria expressing the catalytic-site mutated PgtE D206A did not cleave factors B (Figure 2B) or H (Figure 3B). The catalytic-site substitution D206A rendered also Pla inactive against factor H (Figure 3B). Thus, the breakdown of factors B and H was critically dependent on the direct proteolytic activity exerted by these omptins.

### Mapping of the cleavage sites in H

**Figure 4:**
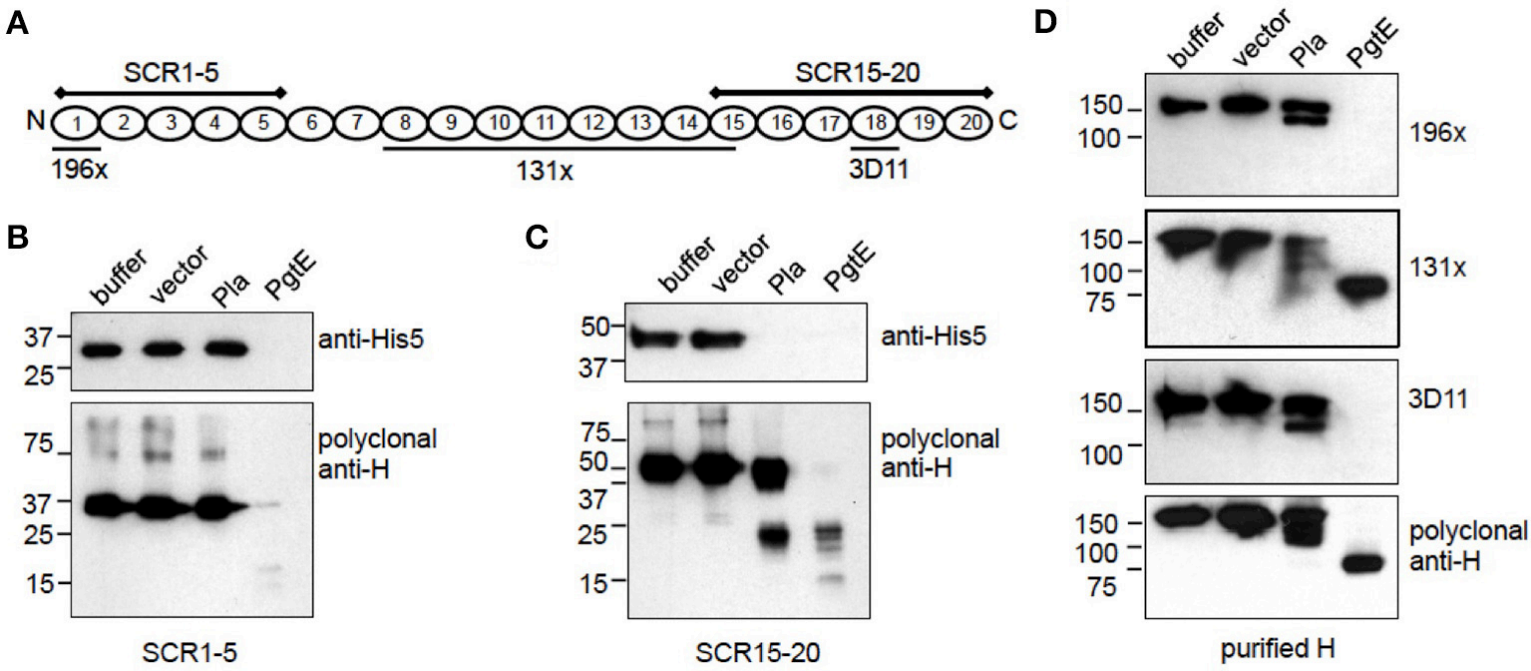
**Mapping of cleavage sites in factor H**. **(A)** Graphic of factor H indicating the areas covered by the recombinant fragments SCR1-5 and SCR15-20 and the localization of the 196X, 131X, and 3D11 mAb epitopes. **(B–D)** *E. coli* 83972 Δ*ompT* bacteria expressing PgtE, Pla or the empty vector were incubated for 1 h with equimolar amounts of recombinant H fragments SCR1-5, SCR15-20 or purified H. The non-reduced supernatants were run in SDS-PAGE and immunoblotted. **(B)** SCR1-5 and **(C)** SCR15-20 blotted with a monoclonal anti-His5 Ab recognizing the C-terminal His8-tag in the H fragments, and with a polyclonal anti-H Ab. **(D)** Purified H blotted with monoclonal Abs 196X, 131X, and 3D11 recognizing the N-terminal SCR1, the SCRs 8-14.5 and the SCR18, respectively; or with a polyclonal anti-H Ab. The assays were conducted at least twice with consistent results.

PgtE and Pla cleaved H into ladder-like patterns indicating cleavages at several sites (Figures 3A,B). H consists of 20 consecutive globular SCR domains connected by short linkers (Makou et al., 2013). To approximate the cleavage sites in H, *E. coli* 83972Δ*ompT* expressing PgtE or Pla were incubated with recombinant, C-terminally His-tagged fragments of H. The areas of H covered by the recombinant fragments SCR1-5 and SCR15-20 are illustrated in Figure 4A. PgtE cleaved the N-terminal fragment SCR1-5 completely during a 1-h incubation, since the epitopes for both the Penta-His mAb and the polyclonal anti-H antibody (H pAb) disappeared. In contrast, Pla did not cleave SCR1-5 (Figure 4B).

PgtE cleaved also the SCR15-20 fragment representing the C-terminus of H, since the intact SCR15-20 detected with either the Penta-His mAb or H pAb disappeared. The H pAb recognized cleavage fragments of ~25 kDa and below indicating several cleavage sites. Pla cleaved SCR15-20 approximately in the middle of the construct and close to either terminus since H pAb recognized a fragment slightly smaller than the intact SCR15-20 and a fragment of ~25 kDa covering ~3 SCRs (Figure 4C). The H pAb, but not Penta-His mAb, recognized also an apparently intact-sized fragment. This could be explained if Pla cleaves off the C-terminal His-tail after SCR20, resulting in the loss of the Penta-His mAb epitope (Kühn and Zipfel, 1995). The amino acid sequence of human factor H terminates in Arg that is a potential P1 residue for omptins (Hritonenko and Stathopoulos, 2007).

Next we continued the cleavage site mapping with purified factor H and monoclonal antibodies targeting different epitopes along H. The mAb 196X recognizes the N-terminal SCR1, the 131X recognition site lies within SCRs 8 through 14.5 and 3D11 recognizes SCR18 as illustrated in Figure 4A. PgtE cleaved H near both termini (Figure 4D) supporting the results with the recombinant SCR1-5 and SCR15-20 fragments (Figures 4B,C), since both the 196X epitope at SCR1 and the 3D11 epitope at SCR18 disappeared. The 131X and pAb recognized an ~85 kDa fragment that represents the central region of H covering approximately 11 SCRs, using the rough estimate of 7.8 kDa per SCR. *E. coli* 83972Δ*ompT* expressing Pla cleaved factor H incompletely in one h since all antibodies recognized the intact H. The antibodies additionally recognized one (196X) or two (131X, 3D11, pAb) smaller cleavage products. The 120 kDa H fragment recognized by 196X, 131X and pAb represented approximately 15.5 of the most N-terminal SCRs. The 112 kDa fragment recognized by 131X, 3D11, and pAb represented approximately 14.5 of the most C-terminal SCRs. This indicated that Pla left the centermost SCRs (approximately 5.5 through 15.5) intact. The cleavage sites were most probably located within linker regions connecting the SCR domains since the non-reduced samples, in which the intra-SCR disulphide bonds remain intact, migrated as separate bands. In conclusion, Pla cleaves shortly after SCR5 and also nearer the C-terminus of H, after SCR15.

### PgtE inhibits opsonization of *S. enterica* 14028R

**Figure 5:**
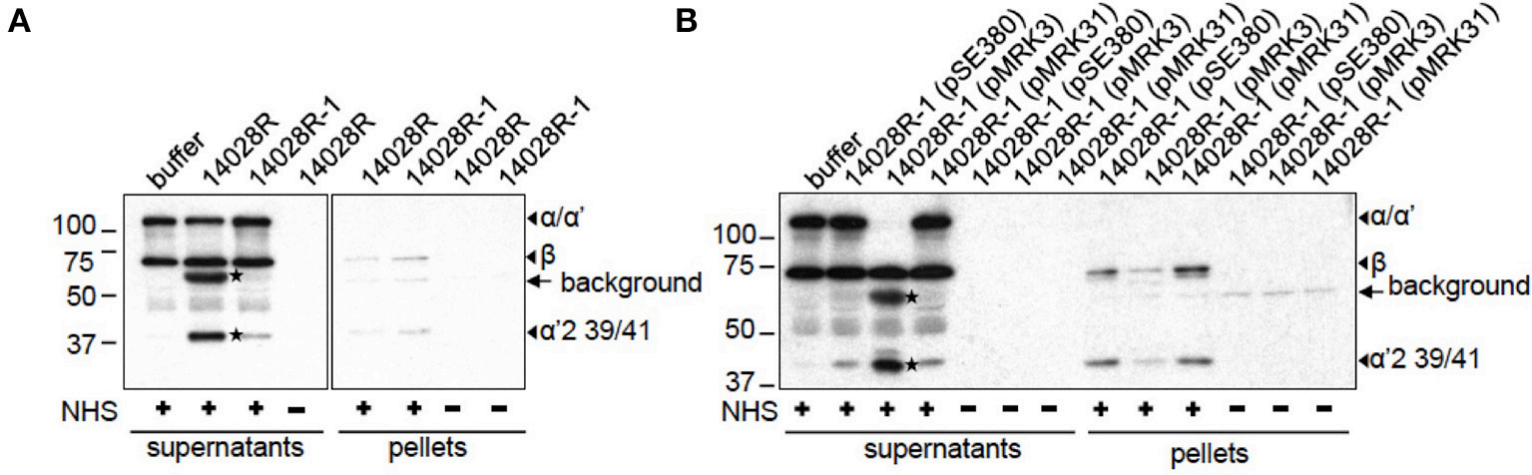
**C3 inactivation by *S. enterica* in serum**. **(A)** *S. enterica* 14028R and 14028R-1 or **(B)** 14028R-1 strains complemented with PgtE (pMRK3), PgtE D206A (pMRK31) or empty vector (pSE380), were incubated in 5% NHS-VBS or in plain VBS for 1 h and pelleted. The reduced supernatants and washed pellets were immunoblotted with anti-C3c and -C3d antibodies. The cleavage fragments of C3 and the non-specific signal from bacteria-originating epitopes are indicated. PgtE-mediated cleavages are marked with asterices (^*^).

We have previously reported that PgtE cleaves purified C3 and C3b (Ramu et al., 2007). However, the cleavage of factors B and H by PgtE could affect the fate of C3 in serum. Firstly, B and H could compete with C3/C3b as substrates for cleavage by PgtE. Secondly, cleavage of H could abolish its complement regulatory activity leading to increased formation of C3b, whereas cleavage of B could lead to fewer active C3 convertases and decreased formation of C3b. Thirdly, PgtE could compete with B and H for C3/C3b cleavage, because Bb in the C3-convertase cleaves C3, and H is a cofactor for C3b cleavage by factor I (Ramu et al., 2007; Torreira et al., 2009; Makou et al., 2013). To test the net effect of PgtE on C3 in the presence of all these factors *Salmonella* strains were incubated in 5% NHS. This serum concentration was chosen to allow an appropriate ratio between the enzyme (PgtE) and the substrates (B, H) for the catalytic reaction. In the human body the bacteria would encounter a whole range of concentrations of B and H because of their diffusion from blood plasma. C3α chain was cleaved into two fragments of ~63 and ~38 kDa when incubated with 14028R (Figure 5A). Notably, the ~63 kDa fragment was not directly PgtE-generated, since PgtE cleaves C3α /α′ into fragments of 46 kDa and smaller (Ramu et al., 2007, and data not shown). Neither does the inactivation of C3b by factor I or plasmin result in a 63 kDa band. However, albumin can affect the migration of nearby bands, thus the ~63 kDa band could be the 68 kDa fragment produced by factor I or plasmin (Barthel et al., 2012)14028R-1 (pMRK3) over-expressing PgtE completely depleted the intact C3α. In contrast, vector- or PgtE D206A-complemented strains did not affect C3α indicating that C3α depletion was dependent on proteolytically active PgtE (Figure 5B). To rule out the possibility that PgtE-expressing bacteria bound C3 fragments and pulled them down, the pellets were examined, too. 14028R-1 (pMRK3) had actually less C3-derived fragments on its surface compared to the vector-carrying strain 14028R-1(pSE380), or to 14028R-1 (pMRK31) expressing PgtE D206A. This indicated that in the absence of proteolytically active PgtE complement activation was directed onto the *Salmonella* surface. Also the activity of PgtE in the presence of all other serum proteins was thus confirmed.

### PgtE interferes with opsonophagocytosis of *Salmonella*

**Figure 6:**
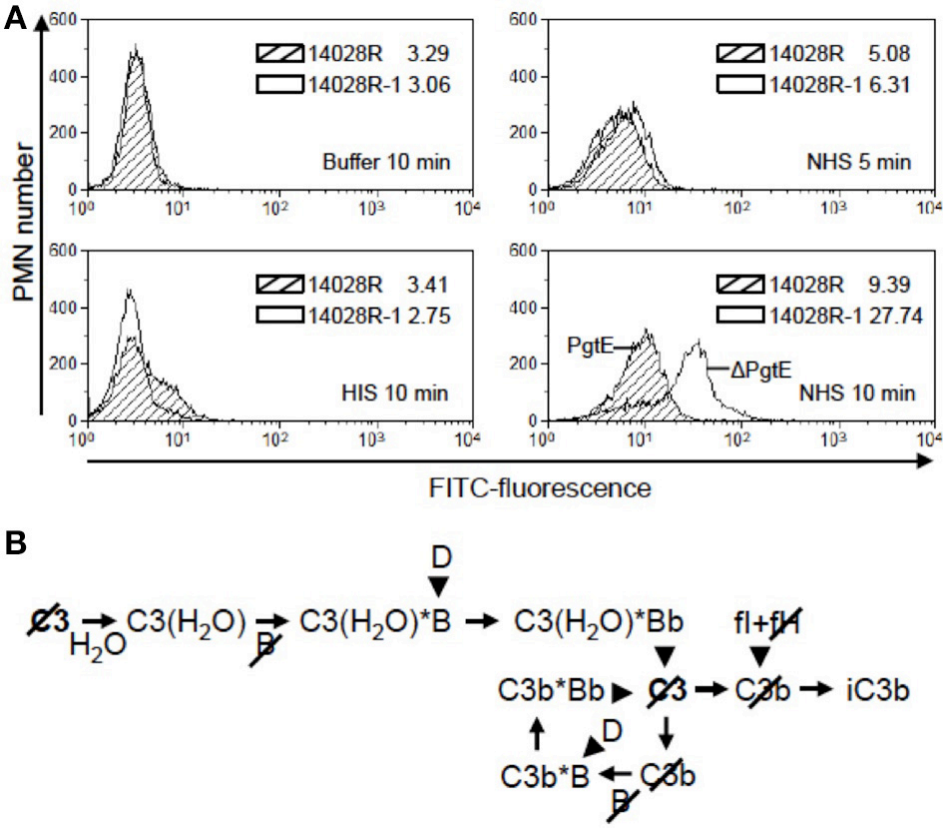
**The effect of PgtE on opsonophagocytosis of *S. enterica***. **(A)** FITC-stained *S. enterica* 14028R and 14028R-1 (Δ*pgtE* derivative of 14028R) were incubated for 5 min in 50% NHS, 50% HIS or buffer. Freshly isolated human neutrophils were mixed with the bacteria at a MOI 20, and phagocytosis was allowed to take place for 5 min (in NHS) or 10 min (in NHS, HIS or buffer). The FITC signals from 10,000 paraformaldehyde-fixed neutrophils were measured using flow cytometry. Median values of fluorescence are shown. Striped area, 14028R; open area, 14028R-1. The experiment was conducted three times, each time with PMN from a different donor and with consistent results. A representative result is shown. **(B)** Progression of the alternative complement pathway up to the level of iC3b that is the main opsonic ligand for CR3 (CD11b/CD18) on neutrophils. Arrowheads indicate proteolytic cleavage. Slashes mark the components cleaved by PgtE.

Since opsonization by C3b or iC3b enhances the uptake of microbes by phagocytes (Ricklin et al., 2010; Bajic et al., 2013), we tested whether PgtE-mediated depletion of C3 from serum would affect phagocytosis. Our experimental setting analyzed collectively both internalized and adherent bacteria. As shown in Figure 6A the fluorescence signal from human PMN incubated with FITC-stained, NHS-opsonized 14028R for 10 min was weaker than that from PMN incubated with *pgtE*-negative 14028R-1. This was not the case when the strains were mock-treated with buffer or HIS suggesting that the effect seen with NHS was dependent on active complement and not on antibodies or direct contact between bacteria and PMN. In conclusion, the expression of PgtE impaired complement-mediated phagocytosis of 14028R.

## Discussion

Microbial proteins that mediate resistance to serum killing or to phagocytosis often recruit complement regulators, such as factor H, onto the microbial surface (Blom et al., 2009; Meri et al., 2013). The proteolytic cleavage of factor B by the virulence factor PgtE of *S. enterica* shown in this study highlights another mechanism of invasive bacteria to resist complement-mediated defense. We also observed cleavage of factor H, which could have a dual effect on complement resistance. As a net effect the degradation of serum complement proteins by PgtE seems beneficial for *Salmonella* survival in the host as it resulted in decreased opsonization and phagocytosis.

*S. enterica* spreads in the host by repeated cycles of phagocyte infection (Mastroeni et al., 2009). Within the phagocytes the activity of PgtE is high, in part due to decreased expression of the O-antigen that inhibits substrate binding by PgtE (Lahteenmaki et al., 2005). On the other hand, O-antigen is a major defense structure of *Salmonella* against complement killing (Frank et al., 1987; Delgado et al., 2006). Our hypothesis is that degradation of B and H by PgtE contributes to the survival of *S. enterica*, when the bacterium is released from one phagocyte and infects another one.

To our knowledge, the only microbial protease that has been reported to cleave factor B to date is an unidentified secreted protease of *Leptospira* spp (Fraga et al., 2014). Factor B circulates in blood as a proenzyme with the scissile bond Arg234-Lys235 between Ba and Bb being protected against factor D-mediated activation. Because the von Willebrand factor type A domain in Bb and the preceding SCR domains in Ba are all involved in binding of B to C3b, and given that Bb, once dissociated from C3bBb convertase, is unable to reassociate with C3b, it is likely that PgtE-cleaved B cannot associate with C3b to form a C3-convertase (Milder et al., 2007; Torreira et al., 2009).

Previously, an unidentified secreted protease of *Aspergillus fumigatus* and the serine protease dentilisin of the periodontal pathogen *Treponema denticola* have been reported to cleave H (McDowell et al., 2009; Behnsen et al., 2010). Recruitment of H by FhbB, the H binding protein of *T. denticola*, facilitates the dentilisin-mediated cleavage (McDowell et al., 2009; Miller et al., 2012). In *Salmonella*, the expression of the H binding protein, Rck,requires heterologous quorum sensing signals (Michael et al., 2001; Ho et al., 2010) and may thereby contribute to PgtE-mediated cleavage.

Factor H is a single-chain polypeptide that folds into 20 globular SCR domains connected by short linkers. Given that the intra-SCR thioester bonds were insufficient to maintain the beads-on-a-string configuration after cleavage by Pla or PgtE, the cleavage of H most probably occurred within the linkers. The proportion of Arg and Lys residues that are preferentially targeted in omptin proteolysis (Hritonenko and Stathopoulos, 2007) is higher in H linkers than in the human proteome on average: 1.7- and 2.6-fold higher, respectively (Lehtinen et al., 2004). This may render the linker regions candidate targets for cleavage by PgtE and Pla. Both PgtE and Pla cleaved H N-terminally to SCR7 suggesting that also FHL-1, the alternatively spliced product of the human *CFH* gene containing H SCRs 1-7 (Estaller et al., 1991) could be cleaved by these omptins.

What could be the functional consequence of the proteolytic attack on H? Cleavage within SCRs 1-5 by PgtE and shortly after SCR5 by Pla separates the cofactor activity located within SCRs 1-4 and the polyanion and microbial binding sites within SCRs 19-20 and 6-7. Thus, the omptin-cleaved H cannot be targeted onto a microbial or host surface to regulate complement activation. Cleavage by PgtE most probably destroys also the cofactor activity itself since SCR domains 1 through 4 are all needed to mediate the cofactor activity in H (Kühn et al., 1995; Makou et al., 2013; Meri et al., 2013). This could lead to more fulminant pathology since the H-mediated protection of host structures would also be compromised. Importantly, the resulting inability of PgtE-cleaved H to control complement activation in the fluid phase would accelerate the depletion of C3 seen with PgtE-expressing *Salmonella*. The local depletion of C3 would efficiently block complement attack against the bacteria.

Although PgtE cleaves complement effector molecules, it has been reported to mediate only modest resistance to direct bactericidal activity of serum (Ramu et al., 2007). Also for *Y. pestis*, Pla is dispensable for serum resistance despite C3 cleavage (Sodeinde et al., 1992; Bartra et al., 2008). Instead, C3 cleavage and interference of complement activation by these omptins could affect phagocytosis since C3b and iC3b are key opsonins. Interestingly, van Bruggen and co-workers showed that the iC3b receptor CR3 (CD11b/CD18) mediated the internalization of *S. enterica* 14028 into human neutrophils (van Bruggen et al., 2007). Neutrophils kill *Salmonella* more efficiently than macrophages (Burton et al., 2014). Here we showed that PgtE expression impairs the interaction, either the adherence or the uptake or both, between *Salmonella* and human neutrophils. We postulate that this is due to PgtE-dependent breakdown of C3 into fragments that have no opsonizing capacity. Importantly, the PgtE-mediated cleavage of C3, H, and B can contribute at several branches in the complement activation cascade to the diminished formation of iC3b, the preferred ligand for CR3, as illustrated in Figure 6B.

In conclusion, this work showed that PgtE cleaves factors B and H thus targeting both the activating and the inhibitory arms of complement. The overall effect of PgtE favors protection of *Salmonella* against the human host immune system, since C3 became depleted from serum, less C3-derived fragments accumulated on *Salmonella* and the association with neutrophils was reduced along with PgtE expression. The results thus suggest that PgtE has an important role in *Salmonella* pathogenesis.

## Materials and methods

### Bacterial strains and plasmids

The previously characterized bacteria and plasmids are listed in Table 1. *E. coli* XL1 - strains were grown, and omptin expression was induced by 5 μM IPTG as previously described (Kukkonen et al., 2001). Other strains were grown with shaking at 37°C overnight, *E. coli* 83972 Δ*ompT* strains and plasmid-complemented *Salmonella* 14028R-1 strains in Luria-Bertani containing 100 μg/ml ampicillin and 5 μM IPTG, and *Salmonella* strains 14028R and 14028R-1 in *pgtE*-inducing media (Lahteenmaki et al., 2005). For C3 cleavage assay the plasmid-complemented *Salmonella* strains were diluted 1:50 with fresh broth in the morning and grown for 2 h further. Bacteria were harvested by centrifugation and washed three times with PBS, pH 7.4–7.5, or veronal buffered saline (142 mM NaCl, 1.8 mM Na-barbital, 3.3 mM barbituric acid, pH 7.4) (VBS).

**Table 1:** **Bacterial strains and plasmids**.

**Strain or plasmid**	**Description**	**Source or references**
***ESCHERICHIA COLI***		
83972 Δ*ompT*	Δ*ompT* derivative of 83972	Järvinen et al., 2013
XL1 Blue MRF'	Δ*(mcrA)183* Δ*(mcrCB-hsdSMR-mrr)173 endA1 supE44 thi-1 recA1 gyrA96 relA1 lac* [F ì *proAB lacI*^q^*ZΔM15* Tn*10* (Tet^r^)]	Agilent Technologies
***SALMONELLA ENTERICA* SEROVAR TYPHIMURIUM**		
14028R	rough LPS derivative of 14028	Wick et al., 1994
14028R-1	Δ*pgtE* derivative of 14028R	Lahteenmaki et al., 2005
**PLASMID**		
pSE380	expression vector, *trc* promoter, *lacO* operator, *lacI*, *bla*	Thermo Fischer Scientific Inc.
pMRK1	*pla* (*Y. pestis*) in pSE380	Kukkonen et al., 2001
pMRK1206	*pla* D206A in pSE380	Kukkonen et al., 2001
pMRK2	*ompT* (*E. coli*) in pSE380	Kukkonen et al., 2001
pMRK3	*pgtE* (*S. enterica*) in pSE380	Kukkonen et al., 2004
pMRK31	*pgtE* D206A in pSE380	Kukkonen et al., 2004
pMRK6	*ompP* (*E. coli*) in pSE380	Järvinen et al., 2013
pMRK8	*ycoA* (*Y. pestis*) in pSE380	Haiko et al., 2010
pMRK9	*ycoB* (*Y. pseudotuberculosis*) in pSE380	Haiko et al., 2010

### Sera, plasma and PMN

Peripheral blood for PMN isolation, for a pool of normal human serum (NHS) and for a pool of plasma for C3 isolation was drawn from consented healthy laboratory personnel. Pooled NHS was aliquoted into single-use batches and stored at −72°C. The NHS pool tested negative for diagnostic *Salmonella* antibodies in O- and H-antigen agglutination tests and in ELISA measuring IgA, IgG, and IgM against LPS of *Salmonella* Typhimurium and Enteritidis. To prepare heat-inactivated serum (HIS), NHS was incubated for 30 min at 56°C. PMN were isolated by Ficoll-Paque PLUS centrifugation as in Jarva et al. (2002) and resuspended in 0.1% BSA-VBS for the phagocytosis assay.

### Complement proteins and antibodies

Factor D was purchased from Merck Millipore. Factors H and B were from Merck Millipore and Quidel Corp. C3 was purified from plasma (Koistinen et al., 1989). H fragments SCR1-5 and SCR15-20 were produced as His-tagged proteins in the baculovirus expression system and purified with Ni-Sepharose (Kühn and Zipfel, 1995). Rabbit antisera against C3c and C3d were from Dako. Goat antisera against B and H were from Merck Millipore.

Monoclonal antibodies 131X, 196X, and 3D11 against H have been described previously (Fontaine et al., 1989; Jokiranta et al., 1996). Penta-His mAb was from Qiagen. HRP-conjugated secondary antibodies were from Jackson ImmunoResearch Laboratories.

### Cleavage of complement proteins

Two parts of bacterial suspension (OD600 = 2 in PBS or VBS) were mixed with one part of protein solution (for H, stock 100 μg/ml; B, 100 or 50 μg/ml; SCR1-5, 25 μg/ml; SCR15-20, 30 μg/ml). Samples were incubated with shaking at 37°C for the indicated times. Bacteria were pelleted at 10,000 g for 3 min. Control cleavage of B by factor D was performed as follows: B (0.5 μg) was incubated with D (0.025 μg) and C3 (1 μg) in 15 μl of 5 mM MgCl_2_-VBS for 1 h. The supernatants (3 parts) were mixed with 1 part of either reducing or non-reducing 4x Laemmli sample buffer (LSB) and heated at 95°C for 3–5 min followed by SDS-PAGE and immunoblotting.

### SDS-page and western blotting

The recombinant H fragments were resolved in 15% SDS-PAGE. For the other samples 10% gels were used. Proteins were transferred onto a 0.2 μm nitrocellulose membrane (Bio-Rad Laboratories). The blocking solution and antibody diluent was 5% skimmed milk/PBS. Enhanced chemiluminescence was detected on Super-RX films (Fujifilm Corporation).

### C3 cleavage in serum

*Salmonella* bacteria (2 × 10^8^ CFU/ml) were incubated in 5% NHS/VBS for 1 h at 37°C with shaking. The bacteria were pelleted, and three parts of the supernatant were mixed with one part of reducing 4x LSB. The pellets were washed with VBS and resuspended in reducing 1x LSB to the original reaction volume. The samples were heated at 95°C for 5 min and run in 10% SDS-PAGE followed by immunoblotting with anti-C3c and C3d antibodies.

### Opsonophagocytosis assay

The phagocytosis protocol was modified from Jarva et al. (2002). Briefly, *Salmonella* strains were grown overnight in *pgtE*-inducing medium and labeled with FITC-Celite® (Sigma-Aldrich) by incubating them for 15 min with shaking at 37°C in 0.5 μg/μl FITC-Celite®/PBS in the dark. The bacteria were washed three times and adjusted to an OD600 value of 0.2 in 0.1% BSA-VBS. Thereafter, the bacteria were incubated in 50% NHS, 50% HIS or plain buffer for 5 min with agitation at 37°C. Freshly isolated PMN were added at a MOI of 20. The PMN were fixed after 5 and 10 min in 2% PFA/PBS for 15 min and run in PBS on FACScan (Becton, Dickinson and Company). The FITC signal was measured from 10,000 PMN and data was analyzed in Summit v4.3 (Beckman Coulter). A comparable FITC-staining of *Salmonella* strains was confirmed using flow cytometry.

## Author contributions

Rauna Riva and Seppo Meri devised the experiments, Rauna Riva conducted the experiments, Rauna Riva, Seppo Meri, and Timo K. Korhonen formulated the manuscript.

### Conflict of interest statement

The authors declare that the research was conducted in the absence of any commercial or financial relationships that could be construed as a potential conflict of interest.

## Abbreviations

AP: alternative complement pathway
B: factor B
H: factor H
HIS: heat-inactivated NHS
NHS: normal human serum pool
pAb: polyclonal antibody
SCR: short consensus repeat
VBS: veronal-buffered saline
